# Georgia Medical Society of Savannah

**Published:** 1871-06

**Authors:** 


### PART IV.

### Editorials, Reviews, Items and News.

### GEORGIA MEDICAL SOCIETY OF SAVANNAH.

We have been furnished a pamphlet containing the report of the delegates of the Georgia Medical Society, and the report of its Committee thereon, in reference to the late action of the Georgia Medical Association held at Americus, in "summarily rescinding" the action of the Association in regard to the Atlanta Medical College, and a portion of its old Faculty, at its annual meetings held at Augusta, 1868, Savannah, 1869, and Macon 1870.

No unprejudiced mind, in reading this pamphlet, can come to any other conclusion than that it is a faithful, impartial history of the unfortunate controversy between the Georgia Medical Association and the Faculty of the Atlanta Medical College, from the time of its introduction into that body. Not one word or sentence in the entire twenty-four pages, can be found, to wound or mortify the old Faculty of the Atlanta Medical College, aside from the faithful record it makes of the controversy. This feature should commend the action of our professional brethren of Savannah to the attentive consideration of the profession of the State. The contrast in tone and kindly language between this Report and that of the "celebrated memorial" is worthy of comment, and furnishes the best evidence of the pure and honorable motives and actions of the old members of the Association in their efforts to give dignity to the body and to uphold the honor and integrity of the profession. Truth never asks the assistance of slander and vituperation in order that it may triumph. It never appeals to personalities to fortify itself. But with quiet dignity, knowing its great power and certain triumph, it points to facts. This is what the Georgia Medical Society has done, and in such a manner as will convince even the enemies of truth that their cause is just and "will prevail."

These remarks are applicable to the members of the Macon Medical Association and all other members of the Georgia Medical Association, who have been of necessity, and not of choice, compelled to take issue with, and pass sentenee upon, the "acts and doings" of the old Faculty of the Atlanta Medical College. So long as the Faculty persisted in its chosen course in defiance of medical law, and in casting reproach upon the integrity and honor of every old member of the Association, no other course of action was left them, however painful the measure, than to pass sentence against it by the severest penalty known to the code —that of expulsion.

The only "animus" pervading the profession against the Atlanta Medical College is that of profound regret that the acts of its Faculty should have made it necessary for the Association to have taken action against it. It is well known that the difficulty in its inception was one entirely independent of the seeking or desire of the professional men of this city, or of the State—one with which they had nothing to do, and for which they can never, in the slightest degree, be held responsible. If a portion of the Faculty, in the face of the plainest teaching of medical law and the usages of all medical schools claiming to be regular, should, at their own option, procure an illegal and unprofessional amendment to the charter, then they, and not the profession, are solely responsible for the "sowing of the seed," and the harvest of difficulties with which they have strewn their path. If this Faculty should assert the right to carry on the institution independent of its Board of Trustees, and seek by legislation to take an institution belonging to the people of the State out of the hands of their legal representatives and place it in the hands of a few professors for their own emolument and professional advancement—then the fault—the sin, if you please—was not that of the Board of Trustees in their worthy efforts to protect the rights of the people, but of the Faculty seeking thus to usurp powers never delegated to them.

And if the Fulton County Medical Society, viewing the whole ground, and appreciating the noble and manly fight for the interests of the people and the profession, should, as honorable professional gentlemen, sympathize both with the people and a Board of Trustees so nobly defending the interests and honor of both, determine to sustain these gentlemen and protect the integrity of the profession by appointing a Committee to carry the matter before the Georgia Medical Association for adjustment and adjudication, whose fault was it? Clearly that of the refractory and rebellious portion of the Faculty of the College, who were seeking to override law, and the guardians of the same; to bring reproach upon the profession, and to lift themselves into power over the heads of its Board of Trustees and the profession of the State. And if they have employed means to carry out these illegal and unprofessional acts, which have brought just reproach upon themselves and their institution, who is to blame for that save the gentlemen themselves.

Can the profession or any member of it compromise with error by ignoring the truth, when such compromise will end in the overthrow and subversion of every principle upon which medical law is founded? There is but one course left the true, manly supporter of principle—that is, to firmly plant himself upon the truth—the whole truth—and never yield an inch to the intrigues and machinations of the supporters of error in the vain hope of combining and mingling in union two antagonistic principles, as an emollient to wounded pride, or the softening of unprofessional hate.

In this connection we call attention to a letter published in another place. The sentiments and advice given the profession in this letter, we fully and heartily endorse. We agree with the author when he so emphatically asserts that "the truth must now be known." This can alone effect a just and final settlement of the issues involved in the controversy, and the truth can alone point the profession to the pathway by which its honor shall be preserved, and full justice done those who are supporters or violators of medical law. He truly states the conclusion of the whole matter by saying, "unless this is done the breach will be widened, and we will all be involved in error—some in one kind, and some in another, and none will be left to correct it." In this way, our friend truly describes the fatal issue of those who would seek to cover up and gloss over the errors of the guilty by half-way measures under the pretentious idea of a so-called compromise. We are glad to know that our friend is "for peace" yet is "unwilling for this difficulty to terminate" in any other manner than upon the principle of truth and justice. Like him, we are unwilling to sacrifice a "single principle, or the character of the humblest brother," to secure "a compromise" at the cost of the true principles which govern, and by its power, throws an egis over the honor and integrity of the profession.

Unless principle is to control, the time is near at hand when truth will find no advocate to uphold it, and error no opposer to condemn it. Truth must be made honorable—so honorable that error will succumb to its power, and be established upon its ruins.

Our correspondent has alluded to some difficulties which we admit. When personal difficulties arise in which no professional conduct or ethical violation, is involved, the profession should not and ough not, to take cognizance of it, but when personal difficulties growout of acts, or anything that affect the profession or any individual member of it, in which the interests of the profession are directly involved, then it becomes the duty of the profession to investigate and settle it, just as was done in the case of Dr. Ramey many years ago. In this instance, only a few members of the Association were affected by his conduct; nevertheless, the Assciation, in view of the insults and slanders heaped upon only a few of its members, in order to vindicate the character of the gentlemen assailed, investigated the matter, and as the result of that investigation, Dr. Ramsey was expelled. Unless this principle is established—unless men are to be supported and, if necessary, honored, when they advocate the truth, what benefit can accrue to them by a connection with organizations? Shall organization be made an engine of oppression, by apologising for the errors of those who defy its power, compromising its members, and the principles upon which its integrity rests? Shall this be done that error may be made respectable, while the truth, and its advocates and defenders are to suffer martyrdom? Then it is better to be in error, and for the first time in the history of the world the old maxim will be reversed, and made to read, "wrong has its rewards," and not "virtue."

So far as we are concerned, we intend to abide the truth—we will never sacrifice it to accommodate error. No sincere lover of truth can, or would, consent to ask an honorable man to yield up even a portion of the truth that thereby error may be met half way and made honorable at the expense of principle. If the old members of the Association have done wrong, let them manly confess the truth and apologize for it. If, on the contrary, they have done right, then those who have done wrong should be made to abide the penalty of their wrong or apologize for it. This is the summing up of the whole matter in a " nut-shell," and any other method of bridging the difficulty will tarnish the reputation of every member, and end in the overthrow of principle and the establishment of error.

We will not be of the number to brand our friends with disgrace, that those who slandered them may be acquitted and justified. Never will we consent to allow, by our act, the charge to be truthfully hurled against us, that for policy or fear, we were forced to submit to the dictates of error, or succumb to the power of its advocates and supporters.

Since the above was in type, we have been put in possession of a pamphlet purporting to be a "statement" of the facts involved in the controversy between the profession of the State and the Atlanta Medical College. We have neither time nor space to make anything like a reply, but desire to assure the profession everywhere, that while the above-mentioned pamphlet does contain some facts, yet the manner in which they are grouped and discussed, makes the document a medley of misstatements and assertions which will, in due time, be thoroughly exposed and fully ventillated. In justice to the old members of the Georgia Medical Association, we ask the profession to await a true history of the whole affair, which will, in a short time, be laid before them.

Our College friends are unfortunate in their selections of persons to do their writing. The "Ransey Sniffle" of this controversy, who, we have reason to believe, wrote the last article headed "A statement of Facts," will, if we are not mistaken, prove to them as troublesome as their unreliable counsel will be expensive. We shall see.

